# A Systematic Review of SBRT Boost for Cervical Cancer Patients Who Cannot Benefit from Brachytherapy

**DOI:** 10.3390/curroncol32030170

**Published:** 2025-03-15

**Authors:** Iozsef Gazsi, Loredana G. Marcu

**Affiliations:** 1 Faculty of Physics, West University of Timisoara, 300223 Timisoara, Romania; jozsi_2007@yahoo.com; 2 County Emergency Clinical Hospital, 410469 Oradea, Romania; 3 Faculty of Informatics & Science, University of Oradea, 410087 Oradea, Romania; 4 Allied Health & Human Performance, University of South Australia, Adelaide 5000, Australia

**Keywords:** stereotactic body radiation therapy, gynecologic cancer, tumor control, toxicity

## Abstract

Stereotactic body radiation therapy has emerged as a promising alternative to brachytherapy, delivering high doses to tumors with precision while sparing surrounding organs. This systematic review evaluates the role of SBRT as a boost for patients who are ineligible for brachytherapy. A total of 17 studies, involving 288 patients, were analyzed, focusing on dosimetric parameters and toxicity. The radiation regimens varied in dose and fractionation schedules, with external beam doses ranging from 44 to 61.6 Gy, and SBRT boost doses ranging from 5 to 30 Gy. The total EQD_2_ doses were between 50.5 and 92.4 Gy. The results indicate adequate tumor control with SBRT, with local control rates ranging from 57% to 95.5%. The acute genitourinary and gastrointestinal toxicities were mostly grade 1 or 2, while late toxicities were less common. The overall survival rates varied between 34% and 96%. These results suggest that SBRT boost offers a viable option for cervical cancer patients ineligible for brachytherapy, with acceptable toxicity and promising survival outcomes. Nevertheless, the scarcity of data, which mainly originate from small studies with patients having varied stages of disease, as well as the lack of long-term follow up with SBRT, should encourage clinicians to utilize brachytherapy whenever suitable as a boost in these patient cohorts.

## 1. Introduction

According to the World Health Organization (WHO) and the World Cancer Research Fund (WCRF) statistics, cervical cancer is the fourth most common malignancy in women worldwide [1]. Low- and middle-income countries from South-East Asia, Central America and sub-Saharan Africa have the highest incidence and death rates from cervical cancer. Inequalities in access to vaccination; screening and treatment facilities; risk factors, such as HIV incidence; and social and economic determinants, such as gender, gender stigma and poverty are all associated with regional differences in the burden of cervical cancer [2].

To achieve high tumor control in cervical cancer therapy, brachytherapy (internal radiotherapy) is an essential treatment component along with external beam radiotherapy, delivering combined doses of more than 90 Gy to the tumor volume. The basic principle behind internal radiotherapy consists of the rapid reduction in peripheral doses while delivering high-dose conformal radiation to the tumor, thereby reducing normal tissue toxicity and optimizing tumor control [3]. Although brachytherapy provides superior protection of neighboring organs at risk, there are several factors that may render a patient ineligible for brachytherapy, such as medical comorbidities, anatomic abnormalities, contraindications to anesthesia or an obstructing tumor mass [4].

The introduction of specialized external radiotherapy techniques has led to a significant reduction in normal tissue toxicities, providing the possibility of administering the whole treatment, including the boost dose, with the same external beam radiotherapy technique, thus justifying the replacement of a brachytherapy boost in ineligible patients [4,5,6,7].

In view of the information above, stereotactic body radiation therapy (SBRT) can deliver a high dose to a tumor volume over a few fractions and can be used for multiple localizations with high accuracy using either on-board imaging or fiducial markers to track both the movement of the tumor volume and the position of surrounding organs [7]. SBRT was found to be suitable for the treatment of several types of cancer, including prostate [8,9], lung [10] and kidney [11]. Additionally, SBRT is now commonly used to treat liver, spine and lung metastases [12].

The aim of this systematic review is to summarize the current literature on the employment of SBRT as a boost for patients with cervical cancer that are either ineligible for or cannot benefit from brachytherapy due to varied reasons. The dosimetric results for tumor control and organs at risk, acute and late toxicities occurring during treatment and overall survival of patients are presented and discussed.

## 2. Materials and Methods

All articles published between 2005 and 2024 that had a reference to SBRT for cervical cancer were included in this review. The databases of Pubmed and the National Library of Medicine were searched using the following keywords: “cervical cancer” OR “gynecologic malignancy” AND “SBRT” OR “SABR” OR “Stereotactic Body Radiation Therapy” OR “Stereotactic Ablative Radiotherapy”.

**Figure 1:**
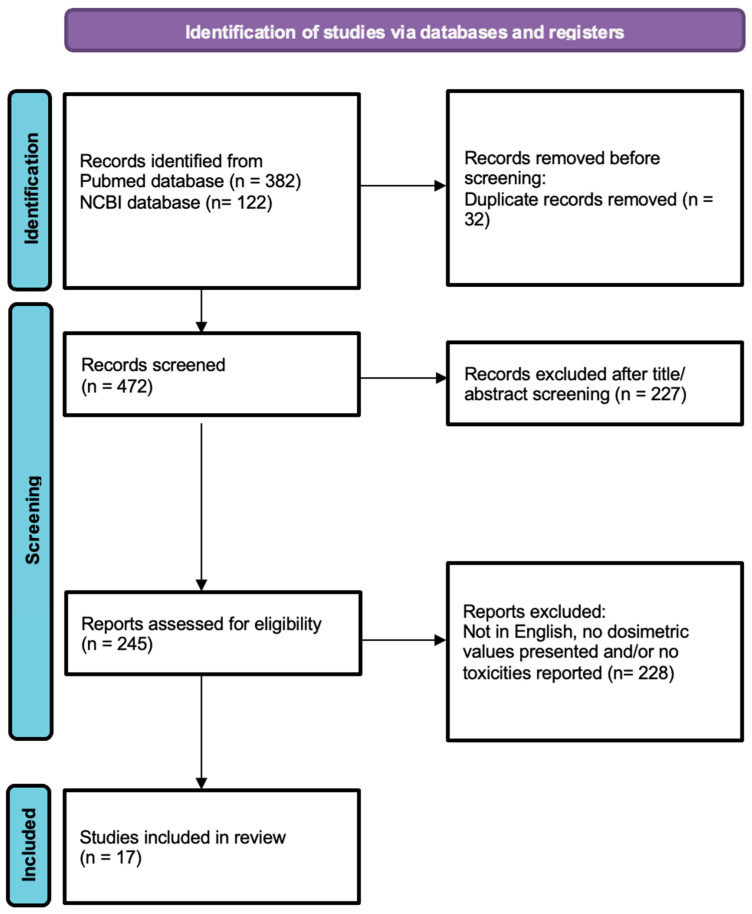
Prisma chart. Study selection diagram.

The eligibility criteria for the selected articles were the following: (1) presents dosimetric parameters pertaining to treatment delivery; (2) reports acute and late toxicities and/or overall survival of cervical cancer patients treated with SBRT/SABR boost. Articles were selected if they presented either separate dosimetric values from boost treatment alone or doses summed with the initial treatment. Articles reporting dosimetric data related to the target volume, as well as for the organs at risk were considered eligible, including articles reporting on acute and/or late toxicities and overall survival. Publications that were not in English, did not report dosimetric values and/or did not report toxicities or overall survival were excluded, as shown in Figure 1. Using the linear quadratic formula with an α/β = 10 for the tumor and α/β = 3 for late effects, the prescribed SBRT dose was converted to a 2 Gy-equivalent dose (EQD_2_) whenever this was not reported by the analyzed study.

The following data were collected from the reviewed articles: number of patients, external dose for primary treatment, SBRT boost dose, equivalent dose, median follow-up time, overall survival, acute and late toxicities (genitourinary and gastrointestinal), tumor volume coverage and doses received by organs at risk.

## 3. Results

A total of 472 studies were screened, of which 17 articles met the eligibility criteria. These studies included an overall number of 288 patients with cervical cancer treated with external beam radiotherapy (EBRT), followed by an SBRT boost with various fractionation regimens.

The studies presented in Table 1 included patients with a wide variety of stages, from early-stage tumors (IA, IB, IIA) to more advanced disease (IIIB, IVA, IVB). The clinical target volume (CTV) and planning target volume (PTV) are determined based on the extent of the disease, with margins ranging from 0 to 15 mm beyond the gross tumor volume (GTV). The GTV is normally determined using a combination of MRI, CT scans and physical examination. The CTV is defined differently according to each individual study: some cover the entire pelvic region or certain regions, such as the vaginal vault, while others concentrate on the cervix, uterus and lymph nodes. Variations in tumor size are reflected in the median tumor and target volumes, which range from 9.163 cm^3^ to 120 cm^3^. Target volume definitions and median tumor volumes, as shown in Table 1, vary owing to different reporting systems used by the evaluated studies. In addition, the information offered by most articles did not differentiate between volume designations for EBRT and SBRT, thus this aspect is reflected in a limited number of studies.

Ineligibility for brachytherapy can occur for a variety of reasons, including anatomical, medical, technical and patient-related factors. Some of the contributing factors are tumor size and location and proximity to vital organs, such as the bladder and rectum, as well as difficulties in positioning the required devices, such as sleeves. Patients with certain medical conditions (cardiovascular, other co-morbidities or poor general health) are ineligible. Finally, patient refusal, combined with factors such as unfavorable anatomy or inability to undergo anesthesia, are other contributors to ineligibility for brachytherapy.

### 3.1. Prescribed Dose and Fractionation: Tumor Volume Dosimetry

**Figure 2:**
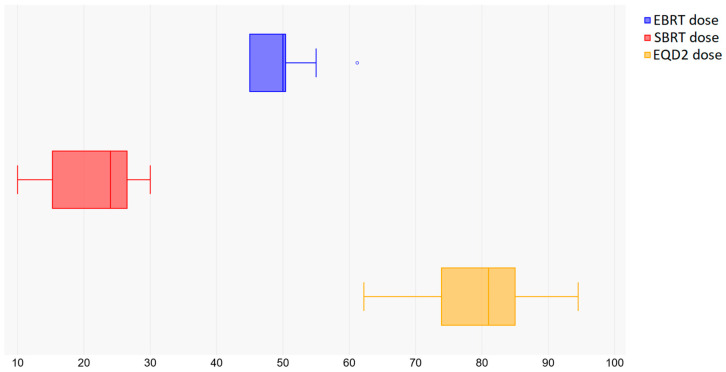
Dose prescriptions for EBRT, SBRT and EQD2.

The doses prescribed for external beam treatment ranged from 44 Gy to 61.6 Gy, with the predominant fractionation schedule being 45 Gy in 25 fractions, as shown in Figure 2. The most common fractionation for the boost volume was 25 Gy in 5 fractions (fr), ranging from 5 Gy/1 fr to 30 Gy/5 fr, with total equivalent doses (i.e., summed with the external dose) ranging from 50.5 Gy to 92.4 Gy for α/β = 10 and from 51.2 Gy to 106.6 Gy for α/β = 3, as shown in Table 2. The median follow-up time varied from 6 to 47 months, and the overall survival ranged from 34% to 96%, depending on years of follow-up. Local control varied from 57% to 95.5%. A Pearson correlation between the total equivalent dose and local control was performed, leading to a weak correlation only, which might be due to the large variations in patient selection, data reporting and follow-up times among the studies.

**Figure 3:**
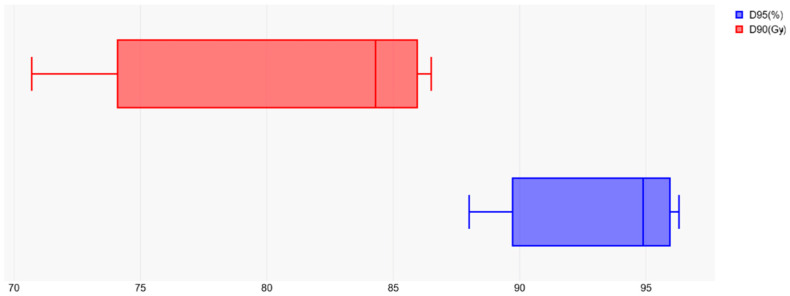
Target volume coverage reflected by D_95_ (%) and D_90_ (Gy) dosimetric parameters.

A dosimetric comparison of the evaluated studies showed satisfactory tumor volume coverage, as illustrated by the collated data of most representative dosimetric parameters (D_90_ (Gy) and D_95_ (%)) presented in Figure 3.

### 3.2. Organ at Risk Dosimetry

**Figure 4:**
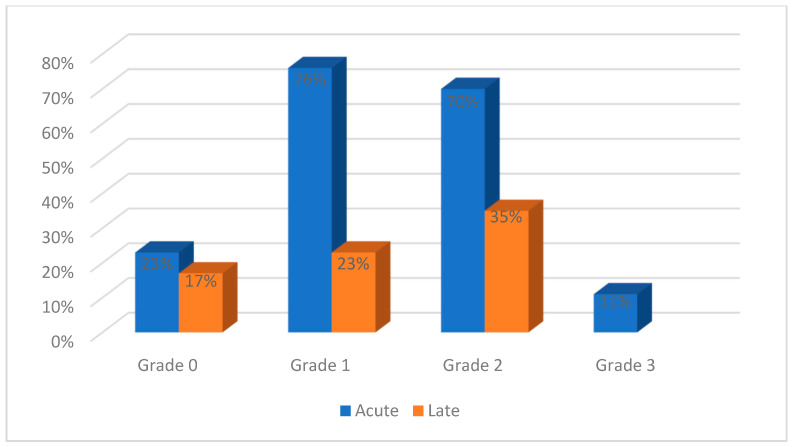
Genitourinary toxicities reported in the evaluated studies.

Acute genitourinary toxicities were reported by most studies and ranged from grades 0 to 3, as shown in Figure 4. Grades 4 and 5 acute toxicities were not reported. Grade 0 toxicities were reported in 4/17 studies, grade 1 toxicities were reported in 13/17 studies, grade 2 toxicities in 12/17 studies, and grade 3 toxicities in 2/17 studies. Late toxicities were presented as grade 0 in 3/17 studies, grade 1 in 4/17 studies and grade 2 in 6/17 studies.

**Figure 5:**
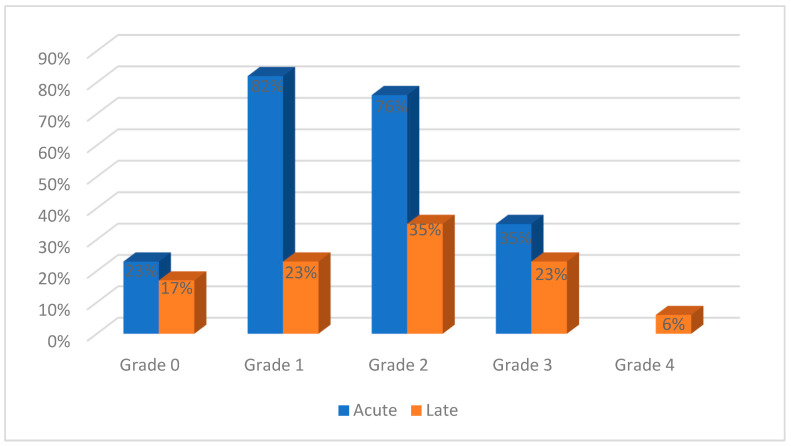
Gastrointestinal toxicities reported in the evaluated studies.

Acute gastrointestinal toxicities were reported in all the studies and ranged from grades 0 to 3, as shown in Figure 5. Grades 4 and 5 acute toxicities were not reported. Grade 0 toxicities were reported in 4/17 studies, grade 1 in 14/17 studies, grade 2 in 13/17 studies, and grade 3 in 6/17 studies. Late toxicities were evaluated in 10 studies, ranging from grade 0 to 4: grade 0 in 3/17 studies, grade 1 in 4/17 studies, grade 2 in 6/17 studies, grade 3 in 4/17 studies, and grade 4 in 1/17 studies.

Regarding bladder dosimetry, only the values presented for the boost volume were different, with Facondo et al. [14] reporting 16.92 Gy for the maximum dose and Pontoriero et al. [15] reporting 17.57 Gy, while the highest value was reported by Kubicek et al. [13] at 25.7 Gy. The lowest value for D_2cc_ was achieved by Pontoriero et al. (3 Gy) [15], and the highest value (19 Gy) was reported by Kubicek et al. [13]. For D_1cc_, Kubicek et al. [13] also reported the highest value (20.7 Gy), whereas Cengiz et al. [29] achieved the lowest dose (6.78 Gy).

The boost dose values summed with the corresponding external beam dose for the bladder are varied. For D_2cc_, the lowest dose was reported by Cheng et al. [19] at a value of 71.97 Gy, and Turna et al. [16] obtained 80.86 Gy. The highest dose was presented by Dahbi et al. [30] at 84.7 Gy.

The rectal doses, according to plans delivered with an SBRT boost only, are diverse. The maximum dose was reported by three studies with very dissimilar values: 8.68 Gy [15], 15.62 Gy [14] and 23.8 Gy [13]. For D_2cc_, the lowest doses were obtained by Pontoriero et al. [15] (4 Gy) and Cengiz et al. [29] (4.56 Gy), while Facondo et al. [14] and Ito et al. [31] delivered larger doses of 13.07 Gy and 16.3 Gy, respectively. The largest dose was again administered by Kubicek et al. [13] (19.3 Gy). Only two studies reported the D_1cc_ parameter: 5.09 Gy [29] and 19.6 Gy [13], respectively.

The combined boost and external beam SBRT resulted in similar doses for the D_2cc_ parameter, ranging from 62.49 Gy [16] to 74.7 Gy [30].

The sigmoid was an under-reported OAR, with a limited number of studies presenting dosimetric values for D_2cc_: 57.65 Gy [16], 62.53 Gy [19] and 75.75 Gy [30].

## 4. Discussion

A number of guidelines recommend that the standard treatment for a patient diagnosed with any type of cervical cancer should be external beam radiotherapy (with or without chemotherapy), followed by 3–4 fractions of brachytherapy with a dose between 5–7 Gy per fraction [32,33,34]. The brachytherapy boost allows the target volume to receive an elevated radiation dose for better tumor control, while sparing the surrounding organs. However, the procedure is invasive and discomforting, usually requiring anesthesia, and employs completely different equipment than external beam radiotherapy, operated by personnel specializing in brachytherapy. It is documented that a small percentage of individuals are unable to receive brachytherapy due to medical comorbidity, anatomical abnormalities or obstructing tumor mass [5].

With the development of specialized radiotherapy techniques, brachytherapy was considered by some to be replaced with SBRT or other external beam techniques. This led to a decline in the utilization of brachytherapy over the first decade of this century (by about 10%), compensated by the increase in the use of EBRT as a boost in cervical cancer [32].

Compared to conventional EBRT, SBRT allows the delivery of a larger biologically equivalent dose to the target in a highly conformal manner over fewer fractions [21,35].

Prior to the widespread use of intensity-modulated and stereotactic body radiotherapy, there were several attempts to replace a brachytherapy boost with the conventional EBRT boost. While the dose delivered to the tumor was often lower than that of the brachytherapy or EBRT boost using contemporary techniques, earlier EBRT boost trials using the 3D-CRT approach showed satisfactory therapeutic outcomes and toxicity profiles [36,37]. Barraclough et al. [4] delivered 60–65 Gy to the tumor volume with conventional fractionation in 71% of patients, reporting good 3-year survival rates (100%—stage I, 70%—stage II and 42% survival among stage III cancer patients), with late grade 1/2 toxicities reported in 41% of patients. These lower doses for target volume coverage were most likely due to the use of fixed fields without intensity modulation by fixed angles, which hindered the reduction in toxicities to surrounding organs at risk.

Ito et al. [38] suggested that an EQD_2_ between 54.2 and 75.3 Gy at point A (the point at which the uterine artery and ureter cross) might result in a 76% pelvic control rate treated with brachytherapy. According to the University of Wisconsin’s experience, a 3-year pelvic control rate of 71% may be attained, with the EQD_2_ = 70.8–84.1 Gy at point A [39]. Likewise, EQD_2_ ranging between 58.3 and 66.7 Gy at point A could offer a remarkable 3-year pelvic control rate (76%) for an advanced stage, according to Toita et al. [40].

Hsieh et al. [18] suggested that an EQD_2_ ranging from 64.6 Gy to 82.7 Gy led to 78% locoregional control with SBRT over a 3-year follow-up, but at the same time, they mentioned that these numbers should be treated cautiously owing to the small number of patients included in their study, as well as the outcome bias induced by the concurrent chemotherapy received by some patients.

Regarding the target volume coverage, Molla et al. [6] used margins of 6–9 mm for PTV in their study but stated that margins of up to 10 mm were more adequate to cover the tumor volume. Jorcano et al. [24] confirmed the need for using a 10 mm margin; they also employed a marker system to compare the CT-based plan with the delivered dose.

Concerning normal tissue toxicity, according to Pourquier et al. [41], the incidence of grade 2 and 3 rectal complications was less than 5% when doses below 75–80 Gy were administered to small-volume tumors; however, the incidence of complications increased to 10–15% with higher doses. To avoid a higher incidence of grade 2+ severe rectal complications, Cheng et al. [42] suggested a proximal rectal dose of less than 62 Gy.

As shown in Table 2, the summed equivalent doses for EBRT and SBRT boost range from 50.5 to 92.4 Gy, but higher doses do not necessarily result in higher toxicities. Pontoriero et al. [15] delivered equivalent doses of 81.5–88.8 Gy and reported both genitourinary and gastrointestinal grade 2 toxicities. On the other hand, Marnitz et al. [22] delivered an equivalent dose of 90 Gy and reported only second-degree toxicities in a similar patient cohort. Dalwadi et al. [25] used equivalent doses up to 89.56 Gy that led to grade 2 late toxicities in only one patient, whereas Park et al. [43] administered doses above 110 Gy and reported higher grade (>2) late genitourinary and gastrointestinal toxicities.

The variability in toxicity outcomes observed across different radiation doses and treatment methods highlights the importance of understanding how different techniques impact both acute and late toxicities. While higher doses in EBRT and SBRT do not always lead to more severe toxicities [44], other factors, such as the proximity of the radiation source to critical organs, play a significant role. For instance, in brachytherapy, where the radioactive source is placed close to the tumor, radiation exposure to surrounding tissues is minimized, potentially reducing toxicity. In contrast, SBRT involves delivering radiation that passes through larger volumes of healthy tissue, which may increase the risk of both acute and late toxicities, particularly in the gastrointestinal and genitourinary systems. This distinction between the radiation delivery methods, along with the dose ranges, contributes to the differing patterns of toxicities observed in the studies discussed.

Table 2 shows that the most commonly reported acute toxicities after SBRT are grades 1 (76%) and 2 (70%) genitourinary toxicities and grades 1 (82%) and 2 (76%) gastrointestinal toxicities. While grade 3+ toxicities are less common, they usually affect the gastrointestinal system. Late toxicities are more likely to be grade 2, both genitourinary and gastrointestinal. Gadda et al. [45] reported one grade 1 acute gastrointestinal toxicity and one late gastrointestinal toxicity of grade 2/3 with brachytherapy, while Chopra et al. [46] reported late grade 2/3 genitourinary (11%) and gastrointestinal toxicity (12.5%) and one patient with grade 4 bowel toxicity from brachytherapy with an EQD_2_ between 80 and 85 Gy.

When comparing the use of SBRT vs. brachytherapy in terms of patient cohorts, Table 3 shows that the number of patients reported in clinical studies employing a brachytherapy boost is larger, owing to the fact that brachytherapy is still the main treatment approach for these patients. However, the staging of disease is comparable between the SBRT and brachytherapy studies, ranging from early stages to advanced ones, with most studies incorporating patients of all stages. Although brachytherapy was implemented in clinics long before SBRT, thus providing more follow-up data, which makes a direct comparison between treatment outcomes more difficult, the evidence to date shows that local control in cervical cancer patients is superior with a brachytherapy boost. Toxicities are generally comparable to an SBRT boost, though in some cases, they may be lower with brachytherapy.

By calculating the equivalent doses for both tumor (α/β = 10) and late effects (α/β = 3) for the two techniques, it can be seen (Table 2 and Table 3) that they are generally within the same ranges. Pötter et al. [47] reported genitourinary and gastrointestinal toxicities in 5.1% of patients after administering equivalent doses of up to 104.4 Gy_3_ with EBRT + brachytherapy boost, with similar outcomes reported by Dalwadi et al. [25] in terms of late toxicities (4%) caused by comparable biological doses (102.4 Gy_3_) delivered via EBRT + SBRT boost. Banerjee et al. [48] concluded that late genitourinary and gastrointestinal toxicities of grade 3 or higher occurring with brachytherapy are less than 10%, while with SBRT, these values are similar for genitourinary toxicities but can go up to 29% for gastrointestinal effects. In the studies shown in Table 3, the percentages of grades 1 and 2 genitourinary and gastrointestinal toxicities are similar to those observed with SBRT, while grade 3 toxicities are lower. This may be due to the fact that with brachytherapy, the radioactive source is placed near the tumor volume, and the radiation does not reach the entire organ at risk, whereas with SBRT, the radiation passes through most of the organs at risk to offer adequate coverage to the tumor volume. The fact that the clinical outcome is generally better with a brachytherapy boost as compared to SBRT suggests that the former remains the main treatment for cervical cancer in patients who are eligible for this type of treatment.

**Table 3:** Clinical outcome after EBRT + brachytherapy boost as reported by the literature.

Study	Number of Patients/Staging	EBRT Dose/Boost Dose Gy (Fr)	EQD_2_ α/β = 10/ EQD_2_ α/β = 3 (Gy)	Median Follow-Up (Months)	Overall Survival	Local Control	Toxicities
Kang et al. [49]	97 IB–IVB	45–50.4 (25–28)/25–30 (5–6)	75.5–87.1/83.2–96.1	50	Progression-free survival: 80% at 3 years	93% at 3 years	2 patients with late rectal bleeding
Castelnaud-Marchand et al. [50]	225 IB1–IVA	44–50.4 (25–28)/15 (3)	62.8–68.3/ 68–72.4	38.8	OS: 76.1% at 3 years	86.4% at 3 years	18 late genitourinary and gastrointestinal toxicities in 14 patients
Simpson et al. [51]	76 IB1–IVA	43.2–50.4 (24–28)/25–30 (3–5)	78.5–89.6/94.3–102.4	17	OS: 75% at 2 years	94.2% at 2 years	2 patients with intractable nausea and vomiting
Charra–Brunaud et al. [52]	117 IB1–IVA	45 (25)/10–20 (2–4)	56.8–69.3/59.2–75.2	24.3	OS: 74% at 2 years	78.5% at 2 years	1.2% of patients with genitourinary and gastrointestinal toxicities
Pötter et al. [47]	156 IB1–IVA	45–50.4 (25–28)/ 28 (4)	83.9–89.2/99.2–104.4	42	OS: 68% at 3 years	95% at 3 years	3 patients with genitourinary and 5 patients with gastrointestinal toxicities
Tan et al. [53]	28 IB1–IIIB	45 (25)/21 (3)	74/85.2	23	N/A	93% at 3 years	2 patients with rectal bleeding

In a study conducted by the Gynecologic Oncology Group, patients treated with chemoradiotherapy (including intracavitary brachytherapy) had a late toxicity rate (grades 3 or 4) of 1.7%, with 22% of patients presenting local progression and an overall survival (OS) of 60% at 5 years [54]. In the study by Chen et al. [55], the use of image-guided brachytherapy demonstrated a 2-year local recurrence-free survival and overall survival of 89% and 85%, respectively. According to O’Donnell et al. [33], patients who received an SBRT boost had OS rates that were equal to those receiving intracavitary brachytherapy. In the articles reviewed, the OS showed a wide variation from 53.3% to 96%, as indicated by studies with a 2-year follow-up, and from 34% to 95% in studies reporting 3-year outcome results. The main reasons for the lower OS are deaths due to multiple metastases and comorbidities. Compared to brachytherapy, those who received an SBRT boost had the same survival rate, while those who received an IMRT boost had a lower survival rate, according to a propensity-matched analysis [33]. Yet, recent results of a phase II trial of an SBRT boost for locally advanced cervical cancer show suboptimal outcomes (53.3% 2-year overall survival), which was justified by patient selection (comorbidities, advanced stage disease) and very large tumors (median PTV size = 139 cm^3^) [23]. The authors only recommend this approach in patients with smaller tumors that are ineligible for brachytherapy.

An important clinical aspect pertaining to SBRT as highlighted by the evaluated studies is the spontaneous organ motion in this anatomical region, leading to interfractional dose variations [56]. For instance, Turna et al. [16] found that for many patients, it was necessary to reposition 2–3 times to achieve the most optimal scenario, including organ filling after performing the position checks. Dincer et al. [57] suggested that a feasible solution to this problem would be the implementation of online adaptive radiotherapy with online contouring and planning. The safety and efficacy of SBRT could be further improved by incorporating tracking techniques, especially for devices with longer treatment times, such as CyberKnife or MR-linac [16].

Despite the disputes regarding the use of SBRT as a boost in cervical cancer management, SBRT is gaining popularity due to accessibility and convenience in use rather than owing to its dosimetric advantage over brachytherapy [32]. The latest investigations across radiotherapy centers, particularly in the United States, point towards underutilization of brachytherapy in cervical cancer patients [58,59]. A survey designed to understand the underutilization of brachytherapy has targeted members of the American Brachytherapy Society (ABS) and showed that the main responsible factors are insufficient training in this field during residency and a lack of professional development to maintain the necessary skills [58].

Nevertheless, brachytherapy is here to stay, and radiotherapy societies and organizations are advised to add more weight to brachytherapy training among radiation oncologists during their residency and to encourage senior clinicians to keep using this technique to improve patients’ outcomes [58]. According to the latest guidelines of the National Comprehensive Cancer Network, brachytherapy is considered a key constituent of cervical cancer management, being pronounced as a critical component of oncological care in patients with unresectable cervical cancers [60]. In view of this, a consensus statement was released by the ABS and the Society of Gynecologic Oncology, whereby external beam radiotherapy, such as IMRT and/or SBRT, should not replace brachytherapy in this patient group [61].

The aim of this review was to evaluate the clinical results of an SBRT boost in cervical cancer in terms of outcome and toxicity and to compare them to conventional brachytherapy boost. This goal was guided by the fact that the number of therapeutic options for patients who cannot or do not wish to receive standard brachytherapy is limited.

This review has a number of limitations, including the large variability among the studies regarding patient-related factors, such as patient selection (particularly variations in staging and tumor size) and patient cohorts (usually small); radiation delivery-related factors, such as different total doses and fractionation schedules (either as a boost or combined techniques); and clinical factors, such as short follow-up times and different reporting of outcomes. Furthermore, the rather limited number of eligible studies and their varied reporting systems renders it difficult to conduct a clinically relevant analysis in order to conclude on definitive judgments about either local control rates or side effect profiles when using SBRT in this patient group.

## 5. Conclusions

An SBRT boost following external beam radiotherapy can be an alternative to brachytherapy in cervical cancer patients who are not eligible for this treatment or refuse it. Although there is a tendency to associate an SBRT boost with high toxicities and a low dose to the target, this technique can achieve target volume doses comparable to those offered by brachytherapy, with acceptable toxicities. It is to be reiterated that the current recommendations towards the use of SBRT in patients with locally advanced cervical cancer include only those situations when patients are ineligible for brachytherapy and should not be employed as a replacement therapy for the latter.

## 6. Future Directions

For situations when SBRT is the only treatment option as boost radiotherapy in cervical cancer, there should be focus on optimizing the dose and fractionation schemes to maximize tumor control while minimizing organ toxicity. Specifically, studies should aim to refine the biologically equivalent dose and EQD_2_ in SBRT, exploring the optimal range for different cervical cancer stages and patient subgroups. To improve treatment protocols, it will be essential to investigate how dose changes in SBRT affects local control, while monitoring acute and late toxicities, particularly in vital organs, such as the bladder, rectum and sigmoid.

Long-term toxicities associated with SBRT compared with conventional brachytherapy, including those affecting the genitourinary and gastrointestinal systems, require further investigation. In addition, research into certain patient risk factors (such as tumor size, HIV infection or other comorbidities) that may confer higher susceptibility to late toxicities will assist in customizing SBRT procedures to reduce these risks.

To address the variability shown in the current review and improve generalizability, international collaborations could standardize procedures and treatment plans in different contexts. These collaborations could potentially enroll larger groups of patients, which would also provide more robust data to support clinical decisions about the use of SBRT in cervical cancer, while still recommending brachytherapy as a key component of cervical cancer management.

## Figures and Tables

**Table 1:** Summary of studies with patient/tumor characteristics and motivations for ineligibility for brachytherapy (N/A = data not available).

Study	Number of Patients and Staging	Target Volume Description/Median Tumor Volume	Reasons for Ineligibility for Brachytherapy
Kubicek et al. [13] 2013	11 N/A	GTV—MRI + physical exam CTV—Whole cervix PTV—5 mm from the CTV Median tumor volume: 9.163 cm^3^	Size and location (1), bleeding (1), proximity to bladder and bowel (1), comorbid conditions (1), difficulty visualizing tumor (1), unable to place sleeve or other problems (6)
Facondo et al. [14] 2021	9 IIA/IIB—3/2 IIIB/IIIC—1/1 IVA—2	CTV—Whole cervix PTV—5 mm from the CTV Median tumor volume: 60.11 cm^3^	Patient refusal (2), cervical stenosis (5), thrombocytopenia (2)
Pontoriero et al. [15] 2015	5 II/IIB—1/2 III—2	GTV—MRI + physical exam PTV—2 mm from the GTV Median tumor volume: 20 cm^3^	Size and location (1), comorbidities (2), proximity to bladder and bowel (1), stenotic and short vaginal stump (1)
Turna et al. [16] 2024	40 IIA/IIB—2/5 IIIC—30 IVA—3	GTV—MRI + PET-CT + physical exam CTV—GTV + Whole cervix PTV—CTV + 5 mm Median tumor volume: 47 cm^3^	Medical (12), technical (13), patient refusal (15)
Haas et al. [17] 2012	6 IIB—4 IV/IVA—1/1	GTV—Whole cervix, with fiducial markers	Anatomy (3), medical (2), patient refusal (1)
Hsieh et al. [18] 2013	9 IIB—4 IIIB—3 IVA—2	GTV- MRI + CT + physical exam CTV—GTV + 7 mm PTV—CTV + 7 mm Median tumor volume: 41.6 cm^3^	To uncannulate the cervical os (4), anaphylactic shock in anesthesia (1), CVA with poor medical condition (1), myoma with contact bleeding (1), old age (1), bed rest with poor medical condition (1)
Cheng et al. [19] 2021	25 IA/IB2—6/3 II/IIB—1/4 IIIA/IIB/IIIC—2/3/2 IVA/IVB—2/2	CTV—Gross disease, superior half of the vagina, presacral region and regional lymph nodes (common, internal and external iliac) PTV—CTV + 8–15 mm Median tumor volume: 26.5 cm^3^	Patient refusal (2), comorbidity (3), fistula (2), laterality of tumor location (4)
Guckenberger et al. [20] 2009	16 N/A	GTV—Residual macroscopic tumor in a planning CT image acquired at the end of the conventionally fractionated irradiation series to account for tumor regression CTV—GTV + 2–3 mm PTV—CTV + 5 mm Median GTV volume: 45 cm^3^ Median PTV volume: 92 cm^3^	Large volume, peripheral location of the recurrent gynecological tumors
Lee et al. [21] 2021	25 I—1 II—10 III—9 IV—5	CTV—All gross tumors, cervix, uterus, parametria and upper vagina CTV nodal—Common iliac, internal iliac, external iliac, obturator and presacral lymph nodes PTV—CTV + 5 mm Median tumor volume: 120 cm^3^	Failure of intracavitary device insertion because of a narrow vagina or cervical os fibrosis (20), large tumor (2), extreme obesity (1), intolerable pain (1), unable to position appropriately due to left spastic hemiplegia (1)
Marnitz et al. [22] 2013	11 IIB—9 IIIB—2	CTV—Whole cervix + MRI PTV—CTV + 0 mm Median tumor volume: 48.9 cm^3^	Uterus bi-collis and bi-cornis (1), patient refusal (10)
Albuquerque et al. [23] 2019	15 IB2–IVB	CTVcervix—Included all gross tumors, the uninvolved cervix, uterus, parametria and 2 cm of vagina beyond the vaginal tumor involvement CTVnodal—Included the common iliac, external iliac, internal iliac, obturator and pre-sacral nodes, with para-aortic nodes if involved PTVnodal—CTVnodal + 7 mm PTVcervix—CTVcervix + 10–12 mm	Medically unfit, patient refusal, tumor extent required IBT for coverage
Jorcano et al. [24] 2010	26 IB/IC—10/7 IIA/IIB—2/5 IIIB—2	CTV1 (primary treatment)—Pelvic lymph nodes +/− para-aortic nodes + tumor bed CTV 2 (Boost)—Vaginal vault, defined as 3 cm of vagina from the vaginal apex PTV—CTV2 + 6–10 mm	N/A
Molla et al. [6] 2005	16 IB2—9 IIB—6 IIIB—1	CTV2 (boost)—Vaginal vault, uterus–parametria, tumor residual or tumor relapse PTV—CTV + 6 or 10 mm	N/A
Dalwadi et al. [25] 2020	25 N/A	CTV—Macroscopic tumor and the remaining cervix PTV—CTV + 5 mm	Medical (9), technical (7), patient refusal (9)
Hadi et al. [26] 2022	10 N/A	CTV—Macroscopic tumor and adjacent areas considered to contain microscopic spread PTV—CTV + 5 mm Median CTV volume: 35.2 cm^3^ Median PTV volume: 43.5 cm^3^	Infiltration on the pelvic wall or on the urinary bladder wall, on the pelvic floor or on the urethra (8), contact with rectosigmoid junction and left ovary (1), not suitable for anesthesia (1)
Kemmerer et al. [27] 2012	11 IA/IB—2/7 IIB—1 IIIC1—1	CTV—A uniform 1 cm expansion around the endometrial cavity and any radiographically identified tumor mass with MRI images	N/A
Lazzari et al. [28] 2020	25 IIB—2 IIIB/IIIC1/IIIC2—3/9/2 IVA/IVB—2/6	CTV—Cervix, uterus, parametria, ovaries, vaginal tissues (based on vaginal extension), involved lymph nodes and relevant draining lymph-nodal groups	Proximity to bladder and bowel (6), unfit for general anesthetic/comorbid conditions (9), isolation/no compliance (5), unfavorable anatomy (5)

**Table 2:** Summary of studies with SBRT boost reporting radiation schedule, EQD_2_, overall survival and toxicities (N/A = data not available).

Study/Year	External Dose (Gy/Fr)	Boost Dose (Gy/Fr)	EQD_2_ α/β = 10/ EQD_2_ α/β = 3 (Gy)	Follow-Up (Months)	Overall Survival (%)/Local control (%)	Toxicity			
						Genitourinary		Gastrointestinal	
						Acute	Late	Acute	Late
Kubicek et al. [13] 2013	45–50.4/25–28	5–27.5/1–5	50.5–80.8/ 51.2–89.95	14	N/A	Grade 2–2 (18%)	N/A	Grade 2–2 (18%)	Grade 3–1 (9%)
Facondo et al. [14] 2021	50.4–61.6/28	15–25/3–5	80.8–92.4/ 88.38–106.6	16	2 years (70%)/N/A	Grade 1–2 (22%)	Grade 2–2 (22%)	Grade 1–1 (11%)	Grade 2–2 (22%)
Pontoriero et al. [15] 2015	45/25	BT: 10–15/2–3 SBRT: 15–20/3–4	81.5–88.8 Gy/ 83.2–92.2	12	1 year/60%	Grade 1–3 (60%) Grade 2–1 (20%)	N/A	Grade 1–1 (20%) Grade 2–1 (20%)	N/A
Turna et al. [16] 2024	45/25	30/5	84.25/97.2	16	N/A	Grade 0–36 (90%) Grade 1–2 (5%) Grade 2–2 (5%)	N/A	Grade 0–37 (92.5%) Grade 1–1 (2.5%) Grade 2–1 (2.5%) Grade 3–1 (2.5%)	N/A
Haas et al. [17] 2012	50.4–61.2/28–34	19.5–20/3–5	76.37–82/ 85.43–86.75	14	N/A	Grade 1–4 (66%) Grade 2–4 (66%)	N/A	Grade 1–4 (66%) Grade 2–4 (66%)	N/A
Hsieh et al. [18] 2013	50–54/25–27	16–27/8–9	65.56–83.25/ 64.38–87.6	36	3 years (46.9%)/77.8%	Grade 1–8 (88%) Grade 2–1 (11%)	N/A	Grade 1–7 (78%) Grade 2–1 (11%) Grade 3–1 (11%)	Grade 2–3 (33%)
Cheng et al. [19] 2021	45–54/25–27	8–30/4–6.5	62.5–89.5/ 85–94	12	N/A/ 80.8%	Grade 1–1 (4%) Grade 2–1 (4%)	Grade 1–1 (4%) Grade 2–2 (8%)	Grade 1–8 (32%) Grade 2–1 (4%) Grade 3–2 (8%)	Grade 2–1 (4%) Grade 3–2 (8%)
Guckenberger et al. [20] 2009	50/25	10–20/ 2–4	62.5–75/ 66–82	22	3 years (34%)/81%	Grade 2–3 (16%)	Grade 2–2 (10%)	Grade 2–3 (16%)	Grade 4–3 (16%)
Lee et al. [21] 2021	44–50.4/22–28	25/5	75.5–80.81/ 84–88.38	34	3 years (77.1%)/ 80.9%	Grade 1–3 (14.3%) Grade 2–5 (23.8%) Grade 3–2 (9.5%)	N/A	Grade 1–6 (28.6%) Grade 2–2 (9.5%)	N/A
Marnitz et al. [22] 2013	50.4/28	30/5	90/102.38	6	2 years (96%)/95%	Grade 1–9 (81.81%) Grade 2–2 (18.18%)	N/A	Grade 1–9 (81.81%) Grade 2–2 (18.18%)	N/A
Albuquerque et al. [23] 2019	45/25	28/4	83.92/99.2	19	2 years—53.3%/70.1%	N/A	N/A	Grade 1–6 (40%) Grade 2–7 (46.66%) Grade 3–6 (40%)	N/A
Jorcano et al. [24] 2010	45–50.4/25–28	14/2	64.1–69.4/ 71.2–76.4	47	3 years/95%	Grade 0–19 (73%) Grade 1–1 (3.8%) Grade 2–5 (19.23%) Grade 3–1 (3.8%)	Grade 0–20 (76.9%) Grade 1–4 (15.38%) Grade 2–1 (3.8%)	Grade 0–6 (23%) Grade 1–11 (42.3%) Grade 2–8 (30.7%) Grade 3–1 (3.8%)	Grade 0–15 (57.69%) Grade 1–7 (26.92%) Grade 2–2 (7.69%) Grade 3–1 (3.8%)
Molla et al. [6] 2005	45–50.4/25–28	14/2 (n = 12) 20/5 (n = 4)	65–75.4/ 73–78.4	18	N/A	Grade 0–12 (75%) Grade 1–1 (6.25%) Grade 2–3 (11.53%)	Grade 0–16 (100%)	Grade 0–9 (56.25%) Grade 1–2 (12.5%) Grade 2–2 (12.5%)	Grade 0–14 (87.5%) Grade 1–1 (6.25%) Grade 3–1 (6.25%)
Dalwadi et al. [25] 2020	45–50.4/25–28	24–30/4–5	76.25–89.56/ 86.4–102.38	25	2 years—(95.5%)/95.5%	N/A	Grade 2–1 (4%)	N/A	Grade 2–1 (4%)
Hadi et al. [26] 2022	50–55/25	10–16/2–4	69.3–83.9/ 66–90.2	9	N/A/90%	Grade 1–4 (40%)	Grade 1–3 (30%)	Grade 1–5 (50%)	Grade 1–4 (40%)
Kemmerer et al. [27] 2012	45/25	30/5	84.25/97.2	10	1.5 years/57%	Grade 1–2 (18%)	N/A	Grade 1–8 (73%) Grade 3–1 (9%)	N/A
Lazzari et al. [28] 2020	45–50.4/25–28	25/5	75.5–80.8/ 83.2–88.38	26	2 years (67%)/78%	Acute genitourinary and gastrointestinal toxicity: Grade 0–15 (60%), grade 1–7 (28%), grade 2–3 (12%) Late genitourinary and gastrointestinal toxicity: Grade 0–16 (67%), grade 1–2 (21%), grade 2–3 (12%)			

## Data Availability

The data presented in this study are available in this article.

## Abbreviations

The following abbreviations are used in this manuscript:

WHO	World Health Organization
WCRF	World Cancer Research Fund
SBRT	Stereotactic body radiation therapy
SABR	Stereotactic ablative radiotherapy
HIV	Human immunodeficiency viruses
EQD_2_	Equivalent dose in 2 Gy fractions
EBRT	External beam radiotherapy
Fr	Fraction
D_95_	The dose received by 95% of the tumor volume
D_90_	The dose received by 90% of the tumor volume
D_2cc_	Minimum dose to the most irradiated contiguous volume of 2cc
D_1cc_	Minimum dose to the most irradiated contiguous volume of 1cc
OS	Overall survival
3D-CRT	Three-dimensional conformal radiation therapy
